# Mutated trimeric RBD vaccines: a platform against variants of concern

**DOI:** 10.1038/s41392-023-01426-3

**Published:** 2023-04-13

**Authors:** Belén Aparicio, Juan J. Lasarte, Pablo Sarobe

**Affiliations:** 1 Programa de Inmunología e Inmunoterapia, Centro de Investigación Médica Aplicada (CIMA, CCUN), Universidad de Navarra, Pamplona, 31008 Spain; 2 Centro de Investigación Biomédica en Red de Enfermedades Hepáticas y Digestivas (CIBEREHD), Pamplona, 31008 Spain; 3 IdiSNA, Instituto de Investigación Sanitaria de Navarra, Pamplona, 31008 Spain

**Keywords:** Medical research, Preclinical research

He et al. recently reported in Nature Communications^1^ the development of a protein vaccine against SASR-CoV-2 Omicron variant consisting of a trimer of the spike protein receptor-binding domain (RBD) of the Delta variant fused to heptad-repeat sequences 1 and 2. This vaccine induced broad- spectrum neutralizing antibodies and fully protected mice and non-human primates against challenge with live Delta and Omicron virus.

Emergence of SARS-CoV-2 variants of concern, especially Omicron, has resulted in an important number of breakthrough infections that may allow viral transmission and generation of new variants. Although initially described as less pathogenic, Omicron has demonstrated an infectious capacity higher than other previous variants. Moreover, sera from vaccinated and convalescent individuals show a poorer recognition capacity against Omicron, due to mutations located at relevant neutralization-associated epitopes. Therefore, development of vaccination strategies affording protection against Omicron is of paramount importance.

With this aim, He et al. recently reported data obtained using a recombinant vaccine based on a trimeric RBD denominated RBD-HR/trimer. The RBD-HR/trimer protein is a typically structure-guided design of a vaccine antigen. It is only composed of the SARS-CoV-2 RBD and HR sequences, and successfully trimerizes RBD without introducing any non-SARS-CoV-2 viral sequences, reserving the native conformation, and readily interacting with the ACE2 receptor. This is important since exogenous sequences in the design could complicate the potential clinical use. Multimeric subunit vaccines have demonstrated superior immunogenicity, therefore, they designed an immunogen with the RBD fused to the heptad-repeat sequences 1 and 2 (HR1 and HR2), that would allow assembling the proteins in a bouquet-like trimeric structure. Moreover, besides providing these structural features without introducing foreign elements, HRs are well conserved between SARS-CoV-2 variants and have been described as targets for neutralizing antibodies. With this structure, He et al. designed a vaccine with RDB sequence obtained from the Delta variant. Previous results had indicated a lower immunogenicity of Omicron-derived vaccines, prompting here the use of Delta as a more immunogenic variant that shares some mutations with Omicron.

Immunization of mice with RBD-HR/trimer vaccine induced antibodies that neutralized pseudovirus and authentic SARS-CoV-2 virus belonging to different variants, including Omicron, at equivalent neutralization levels. Interestingly a three-dose immunization regimen with RBD-HR/trimer or two doses of mRNA plus RBD-HR/trimer induced a superior neutralizing activity than three doses of an mRNA vaccine, suggesting that RBD-HR/trimer could be a suitable booster for individuals previously vaccinated with mRNA vaccines. As for the broad neutralizing capacity of antibodies induced by RBD-HR/trimer, especially recognition of Omicron, authors demonstrate that it is facilitated by inclusion of L452R and T478K mutations, presumably by increasing antigenicity of the vaccine. This potent humoral immunogenicity was accompanied by the induction of strong CD8 and CD4 T cell responses, including memory cells and T follicular helper cells, known to facilitate antibody induction. Finally, in vivo protection assays showed that mice vaccinated with RBD-HR/trimer had undetectable viral load at different organs, associated with normal histological features and lack of inflammation. Similarly, vaccination of non-human primates induced potent neutralizing antibodies against different viruses and protected these animals from infection with a live Delta variant virus.

This study contributes with structural and sequence features necessary to design more immunogenic vaccines against Omicron. Considering structural properties, several multimerization approaches (dimers, trimers or nanoparticles decorated with multiple monomeric proteins) have been used,^2,3^ demonstrating a superior immunogenicity. All these conformations seek to resemble the natural structure found in the SARS-CoV-2 virion, allowing induction of antibodies with higher affinity for the targeted epitopes. A better epitope exposure and a higher Ag receptor cross-linking and signaling have been proposed as mechanisms accounting for this enhanced immunogenicity. However, in addition to this common feature, antigen size (full S or RBD-based immunogens) may dictate differences between these constructs narrowing the epitope repertoire. Considering neutralizing and non-neutralizing antibodies and, although it is difficult to compare their efficacy due to lack of side-by-side studies and to differences in schedules, dose, adjuvants, etc., smaller immunogens are designed to focus on neutralizing epitopes.

On the other side, selection of the most suitable sequence to induce potent Omicron-specific neutralizing responses has been a point of interest, in terms of vaccine immunogenicity and cross-neutralization capacity. There is some controversy in immunogenicity studies, with some authors reporting lower immunogenicity of Omicron-based vaccines, whereas others describe a similar potency. Regarding cross-recognition of Omicron variants by vaccine-induced antibodies, it is dependent on the similarity between the vaccine and the virus targeted. Most Omicron variants are less recognized by antibodies induced by vaccines based on prototype or beta or delta sequences, with a gradual loss of recognition of more recent Omicron variants. Antibodies induced by the vaccine proposed by He et al., based on Delta sequence, nicely recognize and neutralize the original prototype isolate, not differing significantly with recognition and neutralization of beta, delta and Omicron viruses. Similarly, recognition of B.1.1.529 Omicron variant by sera from vaccinated individuals showed a somehow detectable but not dramatic decrease. However, other studies show that, newer Omicron variants XBB or BQ.1, more distant from Delta variant, are barely recognized by sera from vaccinated individuals and mice, suggesting that the closer is the sequence of the immunogen, the better the recognition of the new variant. Thus, although the RBD-HR/trimer vaccine based on Delta variant shows a good neutralization capacity against earlier Omicron strains, the abovementioned results suggest that responses against newer variants might be more affected. However, the RBD sequence of the variant in the RBD-HR/trimer design could be easily changed into the equivalent amino acid sequences of other SARS-CoV-2 variants (e.g., BQ.1, XBB, and etc), leading to a new variant specific vaccine. The self-assembled trimeric-RBD architecture could be developed into a vaccine platform with the capacity of rapidly responding to the currently circulating SARS-CoV-2 variants in the future.

**Fig. 1:**
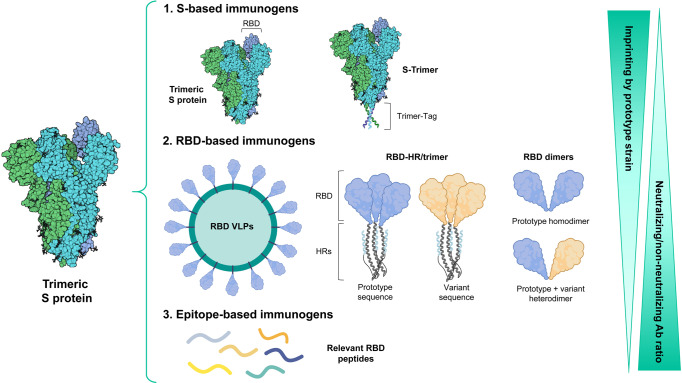
Multimeric vaccine platforms against new SARS-CoV-2 variants. Based on the structure of the natural trimeric S protein, different immunogens have been designed: (1) Native or native-like S trimeric proteins, (2) RBD containing immunogens (multimerized RBD monomers presented on synthetic virus-like particles, RBD-HR/trimer-based vaccines containing trimers of RBD fused to HR1 and HR2 regions from SARS-CoV-2 S protein or RBD homo/hetero dimers), and (3) epitope based immunogens encompassing RBD neutralizing regions. These platforms may have different properties in terms of their immune imprinting from prototype variant and in the induced neutralizing/non-neutralizing antibody ratio (Created in Biorender.com)

Besides results against Omicron obtained after primary immunization, another scenario is the use of new vaccines, including bivalent vaccines, as booster. Clinical studies have shown that the boosting capacity of bivalent vaccines containing prototype sequence plus either BA.1 or BA.4-BA.5 sequence is similar or slightly better than that provided by a monovalent prototype vaccine,^4^ suggesting an important immune imprinting generated by initial antigen exposure. Boosting can be highly biased towards previously encountered shared epitopes, dampening responses to novel epitopes on the newer viral strain. This imprinting might be detrimental when a response to a non-protective epitope dominates. Vaccines with a reduced size, focusing the response on relevant RBD epitopes, as opposed to larger immunogens containing more shared epitopes between variants, might limit the negative effects of imprinting (Fig. 1). Thus, shorter immunogens based on important epitopes, e.g. small proteins like the candidate designed by He at al or peptide vaccines that induce neutralizing antibodies not elicited by the native proteins,^5^ would allow activating new B cell clones specific for the variant sequence not imprinted by original antigenic priming, leading to a higher cross-reactivity against new variants. The strategy proposed be He et al. could be suitable for this approach, by including the adequate sequence according to the predominant variants.
